# Whole Mitochondrial DNA Sequencing Using Fecal Samples from Domestic Dogs

**DOI:** 10.3390/ani14192872

**Published:** 2024-10-05

**Authors:** Takehito Sugasawa, Kieu D. M. Nguyen, Norihiro Otani, Kiyoshi Maehara, Fuka Kamiya, Atsushi Hirokawa, Tohru Takemasa, Koichi Watanabe, Takeki Nishi, Ken Sato, Suzuka Shimmura, Yoichiro Takahashi, Yasuharu Kanki

**Affiliations:** 1 Laboratory of Clinical Examination and Sports Medicine, Department of Clinical Medicine, Institute of Medicine, University of Tsukuba, 1-1-1 Tennodai, Tsukuba 305-8577, Japan; 2 Department of Sports Medicine Analysis, Open Facility Network Office, Organization for Open Facility Initiatives, University of Tsukuba, 1-1-1 Tennodai, Tsukuba 305-8577, Japan; 3 Human Biology Program, University of Tsukuba, 1-1-1 Tennodai, Tsukuba 305-8577, Japan; 4 College of Medicine, School of Medicine and Health Sciences, University of Tsukuba, 1-1-1 Tennodai, Tsukuba 305-8577, Japan; 5 Doctoral Program in Medical Sciences, Graduate School of Comprehensive Human Sciences, University of Tsukuba, 1-1-1 Tennodai, Tsukuba 305-8575, Japan; 6 Institute of Health and Sport Sciences, University of Tsukuba, 1-1-1 Tennodai, Tsukuba 305-8574, Japan; 7 Department of Legal Medicine, Institute of Medicine, University of Tsukuba, 1-1-1 Tennodai, Tsukuba 305-8575, Japan

**Keywords:** domestic dogs, whole mitochondrial DNA, feces, fecal samples, next-generation sequencer

## Abstract

**Simple Summary:**

Several DNA tests are available for individual identification and mitochondrial diseases. Among them, the sequencing of whole mitochondrial DNA (mtDNA) is a beneficial method. Although DNA analysis requires a sample from the individual dog (e.g., blood or tissue), fecal samples are considered non-invasive and the least stressful form of sampling. In this study, we attempted to establish a method for sequencing whole mtDNA using small fecal samples (100 mg) from two domestic dogs. We also attempted to establish a method that is as accessible as possible to veterinary researchers. As a result, we were able to determine the samples’ whole mtDNA sequences with high accuracy. The method developed in this study shows that mtDNA from feces can be decoded and that this form of sampling provides a suitable sample for mtDNA decoding. The presented findings will inform future developments in veterinary medicine and animal welfare.

**Abstract:**

Medical care for domestic dogs is now respected worldwide as being at a similar level to that of humans. We previously established a test method to determine whole mitochondrial DNA (mtDNA) using oral mucosal DNA that may be useful for medical care and welfare. However, the sample types tested in dogs are not limited to those obtained from the oral mucosa. Therefore, in the present study, we attempted to establish a test method to determine whole mtDNA sequences using feces, which represents the least invasive specimen. Two Japanese domestic dogs were used in the present study. DNA was extracted from approximately 100 mg of fresh feces from each dog, and PCRs were performed using four primer pairs that can amplify whole mtDNA. Following PCR, amplicons were pooled to create a DNA library using an experimental robot with an original program. Data were then acquired via NGS and data analysis was performed. The results showed that the whole mtDNA sequence of the two dogs was determined with high accuracy. Our results suggest that feces can be adapted for mitochondrial disease and individual identification testing and could serve as a useful testing method for the future medical care and welfare of domestic dogs.

## 1. Introduction

According to large-scale statistics on domestic dogs in Japan in 2023, 6.84 million dogs are kept as pets and 9.1% of households in Japan share their lives with dogs [1]. Moreover, it is noteworthy that the total number of human children under the age of 15 was approximately 14.17 million, while the number of domesticated dogs and cats in 2023 was 15.91 million, exceeding the number of human children [2]. Against this background, domestic dogs and cats are welcomed as family members just like humans, and are sometimes raised with love in the same way as human children. Reflecting this trend, despite the recent economic downturn in Japan due to the weak yen and other factors, domestic dogs’ expenses, including medical expenses, have increased year on year [3], and the number of contracts with pet insurance companies that cover medical expenses has also increased in the same manner [4]. The above findings indicate that a society is developing in which dogs and cats are respected to a level similar to that of humans, receiving medical care while ensuring that their welfare is looked after.

The medical care and welfare of domestic dogs somewhat lag behind that of humans but are slowly developing day by day and keeping pace with human medical advances. For example, drug treatment options for canine osteoarthritis have evolved over the past decade to keep pace with human medicine [5]. Advances have also been made in the treatment of various canine cancers, which have improved life expectancy [6,7,8]. While canine medicine has evolved, similar to human medicine, there are very few published papers on the development of testing and treatment methods for mitochondrial diseases in dogs.

Canine mitochondrial diseases are caused either congenitally or acquired due to mutations or defects in nuclear DNA, which encodes mitochondria-related genes, or in mitochondrial DNA [9]. Mitochondrial damage resulting from such mutations can severely affect organs that are heavily dependent on oxidative metabolism, especially the brain, skeletal and cardiac muscles, sensory organs, and kidneys [9]. Symptoms of mitochondrial disorders, also known as mitochondrial myopathy, include muscle weakness, gait disturbance, spontaneous pain, and a variety of other symptoms [9]. In addition, it has been shown that canine cancers are associated with mutations in the mtDNA-encoded genetic and D-loop regions [9]. Because these mutations in mtDNA present with a wide variety of symptoms, including mild to severe symptoms, a differential diagnosis is incredibly difficult and requires the development of reliable molecular tests [9].

As a matter of course, in order to develop a treatment strategy for canine mitochondrial diseases, it is first necessary to establish a test method to aid in making an accurate differential diagnosis. In order to make a diagnosis, it is essential to examine the full-length integrity and mutations of mtDNA. Therefore, we previously established a method for resequencing the full length of mitochondrial DNA from canine oral mucosal DNA using a next-generation sequencer (NGS) to accurately decode sequences and mutations [10]. This method may be of great help in the differential diagnosis of canine mitochondrial diseases. It could also be applied to identify individual dogs because the mammalian mitochondrial genome is maternally inherited. Nevertheless, it is recognized that many dogs show a lack of willingness to have their oral mucosal DNA collected. Moreover, the biological samples that can be collected from dogs are not limited to oral mucosal DNA. Various types of samples can be collected, including blood, skin tissue, urine, hair follicles, residual tissue after surgery, feces, etc. Therefore, we focused our attention on feces, which can be easily collected in the least invasive manner as a lightweight solid material.

Feces are a mixture of undigested food residues, intestinal bacteria, cholesterol, and fats [11,12]. They also contain cellular debris detached from the intestinal mucosa and dead white blood cells [11,13]. In addition, mammalian cell mtDNA copy numbers have been shown to be present in hundreds to thousands of copies per cell, although this depends on the specific tissue [14,15]. In other words, canine feces may contain high levels of mitochondrial DNA. In light of the above, it could be hypothesized that the whole mtDNA of dogs can be sequenced and decoded using DNA extracted and purified from feces. Therefore, the purpose of the present study was to establish a method to decode the whole mtDNA sequence using DNA obtained from canine feces.

As a result of the development of our method, the whole mtDNA sequence from the feces samples was successfully decoded with high accuracy. It is anticipated that this method will be utilized for the individual identification of dogs and the differentiation of mitochondrial diseases, which may prove advantageous for the future medical care and welfare of domestic dogs.

## 2. Materials and Methods

### 2.1. Informed Consent

All owners who participated in the study were informed about the design of the study and their consent was obtained.

### 2.2. Subject Dogs and Collected Feces

**Figure 1:**
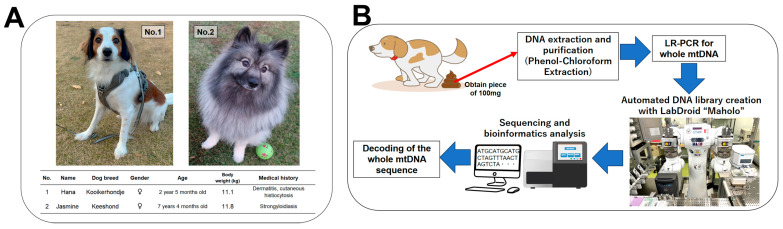
Characteristics of the two dogs in the present study and a summary of the experiment: (**A**) exterior view and characteristics of the two dogs, and (**B**) overview of experiments for whole mtDNA sequencing. LR-PCR: long-range polymerase chain reaction.

Two Japanese domestic dogs were enrolled in the present study. The breeds were Kooikerhondje and Keeshond. The age, medical history, weight, and other characteristics of the two dogs are shown in Figure 1A together with photographs showing their appearance. Once each dog had defecated naturally, the feces were sealed in polyethylene bags at natural temperature and transported to our laboratory. Small pieces of feces (approximately 100 mg) were prepared within 1 h of defecation and used for DNA extraction experiments. An overview of the fecal collection process and subsequent experimental steps is shown as a schematic in Figure 1B.

### 2.3. DNA Extraction from the Feces

Fecal DNA was extracted and purified using a method previously reported by our research group [10]. Approximately 100 mg of feces was homogenized in 500 μL of SNET buffer (10 mM Tris, pH 8.0; 100 mM EDTA, pH 8.0; 1% SDS; 100 µg/mL Proteinase K) using crushing beads and bead crusher apparatus. The homogenate was then incubated at 56 °C for 1 h to degrade the protein. Following incubation, 500 μL of phenol/chloroform/isoamyl alcohol (25:24:1) solution (Cat# 25970-56; Nacalai Tesque, Nakagyo, Kyoto, Japan) was added and shaken vigorously for 15 s. The mixture was allowed to stand at room temperature for 10 min. The mixture was centrifuged at 12,000× *g* for 15 min and the supernatant, which is the aqueous layer, was aliquoted at 300 μL into new microtubes. The 300 μL aliquot was added to ribonuclease solution (at a final concentration of 10 ug/mL) and then incubated at 37 °C for 10 min. Following this incubation procedure, the solution containing DNA was purified from the mixture using a NucleoSpin Gel and PCR Clean-up kit (Cat#U0609B; Takara Bio, Kusatsu, Shiga, Japan) in accordance with the manufacturer’s manual. Lastly, the DNA was eluted in 50 µL of Tris-HCl buffer (5 mM Tris, pH 8.5).

### 2.4. Analysis of DNA Integrity

The concentration of the purified DNA was determined using a NanoDrop Lite spectrophotometer (Thermo Fisher Scientific, Waltham, MA, USA). Furthermore, to assess the integrity of the DNA, in particular, to examine any degradation or fragmentation, the purified DNA was analyzed using an Agilent 2100 Bioanalyzer system (Agilent Technologies, Santa Clara, CA, USA) coupled with an Agilent DNA 12,000 Kit (Cat#5067-1508; Agilent Technologies). Moreover, intact mouse liver DNA was used as a positive control (intact DNA) during this analysis [16].

### 2.5. LR-PCR for Whole mtDNA

Long-range polymerase chain reaction (LR-PCR) was performed according to our previously reported method [10]. This LR-PCR, using four primer pairs, can amplify the full length of mtDNA [10]. A summary of each primer sequence and target location is shown in Supplementary Table S1 with sequences of the primer pairs. The information shown in Table S1 is used with reference to our previous study [10]. First, to determine the optimal amount of template DNA, DNA from dog No. 1 was used, and serial dilutions of the template DNA were ranged from 0.01 to 100 ng/μL. The LR-PCR was performed using KOD One PCR Master Mix reagent (Cat# KMM-101; TOYOBO, Osaka, Japan), including a high-fidelity PCR enzyme. The template and reagent volume and primer concentrations were 2 and 10 µL and 300 nM, respectively, for a total reaction volume of 20 µL per reaction tube. A positive or negative control sample was also prepared using cell line DNA from the dog (1 ng/µL) or Milli-Q water. The conditions for thermal cycling were as follows: 98 °C for 1 min, five cycles of 98 °C for 10 s and 74 °C for 30 s, five cycles of 98 °C for 10 s and 70 °C for 30 s, five cycles of 98 °C for 10 s and 72 °C for 30 s, 30 cycles of 98 °C for 10 s and 68 °C for 30 s, and 4 °C for ∞ [10]. The size of the amplified products was electrophoresed in the same manner as described in Section 2.4. to confirm specificity and optimal template concentration. As it was determined that 10 ng/µL was the optimal template DNA concentration in the experiment thus far, LR-PCR was repeated with the DNA concentrations of dogs No. 1 and No. 2 (NucleoMag NGS clean-up and Size Select; Takara Bio) set at 10 ng/µL to obtain the desired amplified product. Thereafter, the amplified products were electrophoresed once again, and the concentration (nM) of the amplicon of interest was calculated and pooled so that each of the four amplicons was identical in terms of nM concentration. Following pooling, the amplicons were purified using 0.8 × beads (NucleoMag NGS clean-up and Size Select; Takara Bio) and eluted at 10 µL. The concentration of purified amplicons was measured with a nanodrop, adjusted to 10 ng/µL, and used in the subsequent experiment.

### 2.6. Preparation for DNA Library and NGS Run

Libraries were created for sequencing via NGS using adjusted and pooled amplicons. The library was created using 50 ng of pooled amplicon DNA. The reagent used to create the library was NEBNext Ultra II FS DNA Library Prep Kit for Illumina (Cat# E7805S; New England Biolabs, Ipswich, MA, USA). An automatic experiment processing program that conforms to the protocol of this kit was established on LabDroid “Mahoro” (experimental robot; RBI, Koto City, Tokyo, Japan) to automate library creation (Figure 1B, lower right). The fragmentation process time was set to 6 min in the protocol. In addition, 200 ng of fecal DNA not subjected to LR-PCR was used as a negative control sample, and the libraries were prepared using the same method. The created DNA library was analyzed using an Agilent 2100 Bioanalyzer system (Agilent Technologies) with the Agilent DNA 7500 Kit (Cat#5067-1506; Agilent Technologies). Thereafter, the libraries were adjusted to 10 nM and pooled in an equal volume into one tube.

The pooled libraries were mixed with the PhiX Control v3 Library (final 10%; Cat# FC-110-3001, Illumina, San Diego, CA, USA), diluted to 1 nM, and then subjected to denaturation and neutralization processes. Subsequently, the libraries were diluted further to 1.4 pM and then applied to an NGS run using a MiniSeq Mid Output Kit (300 cycles) (Cat#FC-420-1004; Illumina) in the MiniSeq System (Illumina). Sequencing was performed with the paired-end reads of 150 bases. The cluster density was 264 K/mm^2^ in the MiniSeq System. In addition, the passing filter rate of over Q30 for the clusters was 91.36%, and the data yield was 5.15 G bases with 34.08 M paired-end reads in the equipment. In addition, the mean quality score was 34.99. Overall, the NGS run was considered capable of obtaining high-quality data. Subsequently, FASTQ files were exported following the performance of further bioinformatics analyses.

### 2.7. Bioinformatics Analysis to Determine Whole mtDNA Sequence

The basic information of the NGS run data was confirmed with CLC Genomics Workbench 24.0 software (QIAGEN, Hilden, Germany). During quality assessment of the reads, a PHRED score of over 20 was confirmed for 99.8% of all reads, indicating the success of the run. The read numbers in the FASTQ files were 7.2 to 9.1 M per sample as paired-end reads. The resequencing analysis for the mtDNA was performed following procedures performed using CLC software. First, because the number of reads obtained on this occasion was predicted to be too high, we used the tool “Subsample Sequence List” to adjust all samples to 1 million reads on the FASTQ data. Thereafter, mapping was performed using the tool “Map Reads to Reference” with the general whole mtDNA sequences of the dogs [17] to create BAM files. Subsequently, FASTA files including the whole mtDNA sequence were extracted using the tool “Extract Consensus Sequence” with a quality score. In addition, the tool “Alignments and Trees” was used to analyze whether the mtDNA sequence obtained from the feces was an exact match to the mtDNA sequence that we had previously determined from the oral mucosal DNA [10]. Moreover, to visualize the uniformity of the mapping to the reference sequence, the tool “Create Track List” was used, and the figure included each sample and mitochondrial gene region.

## 3. Results

### 3.1. DNA Integrity

**Figure 2:**
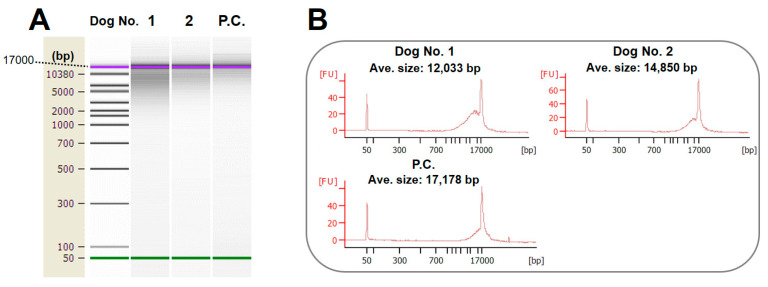
Confirmation of DNA integrity: (**A**) electrophoresis diagram, and (**B**) histogram based on electrophoresis data. P.C.: positive control (intact DNA).

The average size of DNA extracted and purified from the feces of the two dogs was 12–14 kbp. In contrast, that of DNA extracted from intact mouse livers was 17 kbp (Figure 2A,B). Moreover, severe fragmentation was not observed. The above results indicate that the DNA in the dogs’ feces was slightly degraded. However, it was considered of sufficient quality to be used in the subsequent experiment.

### 3.2. Optimization of LR-PCR

**Figure 3:**
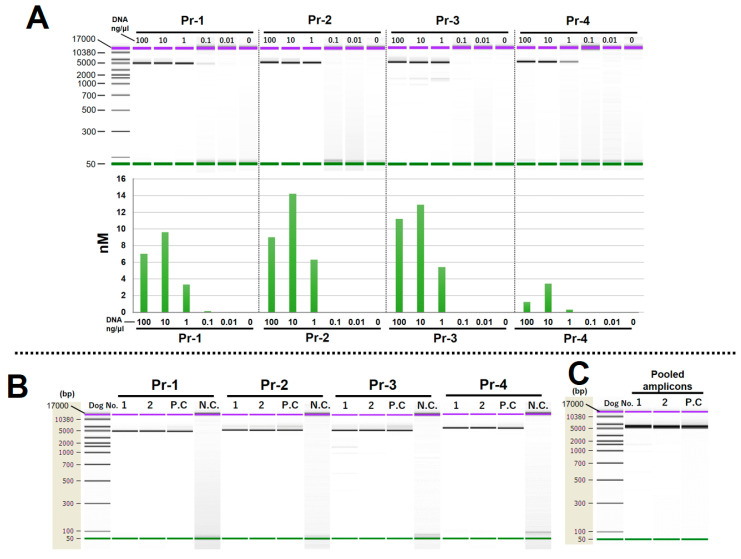
Optimization of LR-PCR and the obtained amplicons: (**A**) LR-PCR using DNA from serial-diluted feces DNA as a template on dog No. 1; (**B**) obtained amplicons from LR-PCRs for the DNA of dogs No. 1 and 2. (**C**); electrophoretic picture of equally pooled amplicons. Pr: primer pair, P.C.: positive control (cell line DNA of the dog), N.C.: negative control (Milli-Q water).

In LR-PCR with serial-diluted fecal DNA, a concentration of 10 ng/µL showed the highest amplification efficiency (Figure 3A) on all primer pairs. The same LR-PCR procedure was then performed with DNA from the two dogs adjusted to the same concentration (10 ng/µL). In the results, amplified products were obtained with sufficient specificity on all primer pairs (Figure 3B). The pooled amplicons were purified, and the amplicon size was confirmed via electrophoresis, which revealed no issues and confirmed that the amplicons were pooled correctly (Figure 3C).

### 3.3. The DNA Libraries Were Accurately Created by the LabDroid “Maholo”

**Figure 4:**
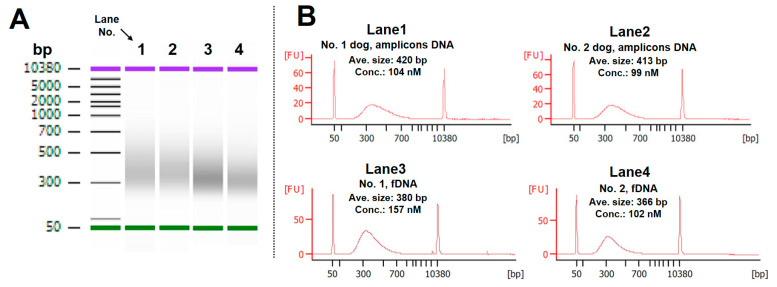
DNA library created by the LabDroid “Maholo”: (**A**) electrophoretic diagram of its library. Lane1: library using the amplicons of dog No. 1; lane2: library using the amplicons of dog No. 2; lane3: library using the fecal DNA of dog No. 1; lane4: library using the fecal DNA of dog No. 2. (**B**) Histogram, average size, and concentration based on the electrophoresis data. fDNA: fecal total DNA.

The LabDroid “Maholo” was used to create DNA libraries using pooled amplicon DNA and fecal DNA. As shown in the results, DNA libraries ranging from 366 to 420 bp in average size were obtained, and this size range is considered as fully adaptable for sequencing (Figure 4A,B). The above result suggested that the LabDroid “Maholo” performed the experimental operation accurately.

### 3.4. The Whole mtDNA Sequence of Both Dogs Was Accurately Determined

**Figure 5:**
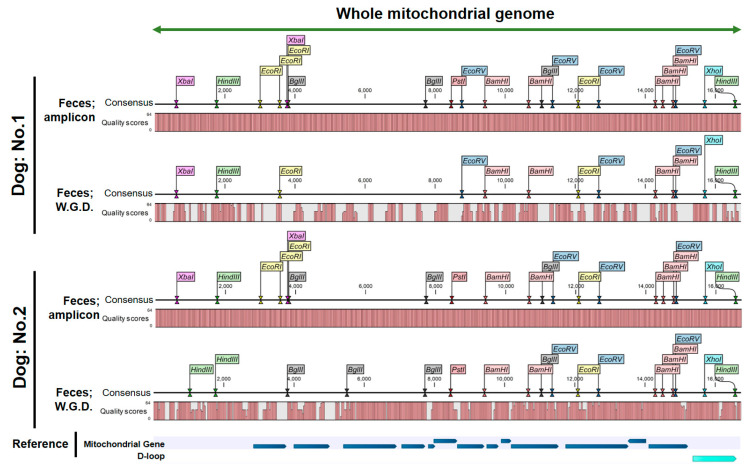
Extracted whole mtDNA sequences with quality scores and restriction enzyme sites. Holes in the bar plot indicate a poor-quality score; i.e., the correct sequence was not obtained. W.G.D.: whole genome DNA.

A consensus sequence was extracted from the BAM file following mapping based on the FASTQ data derived from the amplicons. As a result, the highest quality score of 64 points was observed at all base positions, and the whole mtDNA sequence of the two dogs was accurately determined (Figure 5). In comparison, mapping using BAM files based on fecal DNA did not yield an accurate mtDNA sequence with a poor-quality score (Figure 5). The above results indicate that LR-PCR is essential to accurately determine the whole mtDNA sequence.

### 3.5. The Amplicon Sequencing of mtDNA Using Fecal DNA Provides Uniform Coverage and Accurate Sequencing

**Figure 6:**
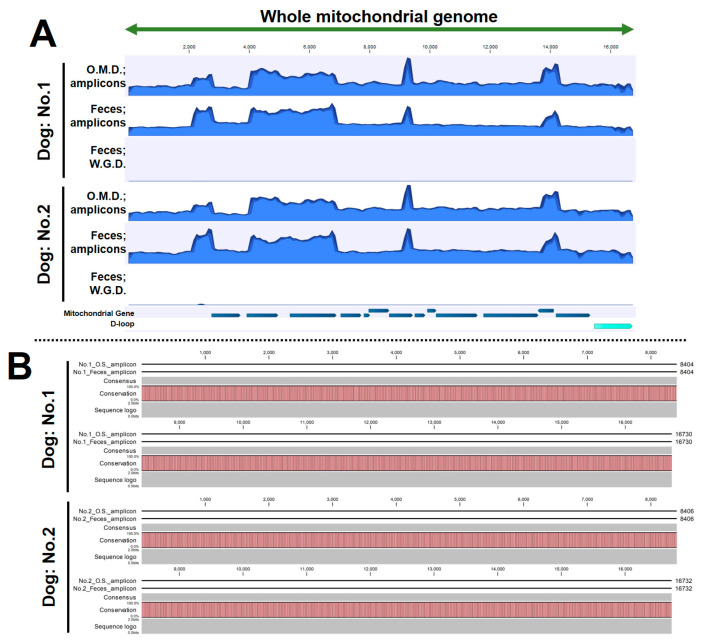
Coverage waveform through visualization of BAM files and alignment of nucleotide sequences: (**A**) coverage waveforms using the BAM files from each sample, and (**B**) alignment analysis between amplicon sequencing of fecal DNA and oral mucosal DNA. This figure is useful for visually checking for mismatched bases. In the event of mismatches in the base sequences obtained from oral mucosal DNA and fecal DNA amplicon sequences, the pink is not displayed, and a white blank is displayed instead. Therefore, the absence of a blank can be taken to confirm that there is a complete match on the alignment analysis. O.M.D: oral mucosal DNA, W.G.D: whole-genome DNA.

BAM files derived from the fecal DNA amplicons were visualized on the reference sequence. As a result, uniform coverage was observed in both dogs, including four overlapping locations. (Figure 6A). Amplicon-derived BAM files of oral mucosal DNA were also visualized [10], as previously reported by our research team, and similar coverage to that of the fecal DNA amplicons was found (Figure 6A). However, the BAM file based on fecal DNA not subjected to LR-PCR failed to show a waveform of coverage. In addition, a comparison of sequences obtained from amplicon sequencing of fecal DNA (Supplementary Data S1 and S2; FASTA files) with those obtained from amplicon sequencing of oral mucosal DNA [10] showed complete match across all bases in the two dogs (Figure 6B). The above results suggest that amplicon sequencing using fecal DNA can determine whole mtDNA sequences as accurately as that using oral mucosal DNA.

## 4. Discussion

The objective of the present study was to develop a method for determining the whole mtDNA sequence of domestic dogs using the least invasive method available. In addition, we used our previously successful sequencing data of whole mtDNA by using oral mucosal DNA [10] and compared them to the data from the fecal samples in the present study to confirm their accuracy. As a result, we succeeded in obtaining whole-length mitochondrial DNA amplicons divided into four parts from fecal DNA. Library preparation using the amplicons was automated using an experimental robot, and we were able to obtain a library that can be adapted to next-generation sequencers. Through bioinformatics analysis using the data obtained from sequencing, we were able to accurately determine the mtDNA sequences derived from the feces of the two dogs. Furthermore, this mtDNA sequence was a perfect match to the sequence derived from the oral mucosal DNA. Taken together, these results indicate that the whole mtDNA sequence can be determined from fecal samples using the method established in the study presented herein. Feces represent the least invasive form of sampling and their acquisition does not cause stress to the dog. Therefore, in the future, the use of this method could lead to advancements in the medical care and welfare of dogs.

The use of this method yields four PCR amplicons containing the whole mtDNA sequence (including four duplicated regions), which can be sequenced using a next-generation sequencer. This principle is similar to our previously established method for whole mtDNA sequencing using oral mucosal DNA [10]. However, we found one difference: in PCR using oral mucosal DNA, the highest amplification efficiency was observed when the template DNA concentration was 1 ng/µL (using 2 ng DNA/PCR reaction) [10]. Conversely, when fecal DNA was used as a template in the present study, the highest amplification efficiency was achieved at a concentration of 10 ng/µL (using 20 ng DNA/PCR reaction). This difference is likely due to the high mix of DNA from different species in the fecal material, which may comprise food residue and bacteria. Bacteria in fecal solids, excluding water, are estimated to account for 30% to 50% of fecal material [11,13]. Therefore, depending on the type of specimen, the template concentration should be optimized. In addition, although the two dogs in the present study were healthy on the day of the experiment, if the subject dogs in a study suffer from intestinal conditions/diseases such as diarrhea, constipation, inflammatory diseases, etc., the percentage of intestinal bacteria may vary substantially. If researchers use this method, they would need to first consider the optimal template DNA concentration to adapt to this situation.

The establishment of a non-invasive method of sequencing mtDNA from feces has the potential to promote the understanding and management of mitochondrial diseases in domestic dogs. Multiple mitochondrial diseases, such as myopathies, sensory ataxic neuropathy, cardiomyopathy, and arrhythmia, have been reported in domestic dogs and clinical symptoms are heterogeneous, causing difficulties in diagnosis. Many pathogenic mtDNA mutations have been identified in dogs; thus, it is possible to diagnose mitochondrial diseases through genetic evaluation [18]. To date, mitochondrial diseases are rarely found in domestic dogs. However, the significant health impact and frequency of human mitochondrial mutations combined with the genetic similarity between humans and dogs suggest that mitochondrial DNA alterations could also have a significant health impact on domestic dogs and the prevalence of mitochondrial diseases in dogs could be underestimated due to insufficient investigation. Thus, it would be beneficial to perform mitochondrial genetic analyses in veterinary clinics to better understand the genetic causes of diseases, consequently revealing the frequency of mitochondrial diseases. mtDNA genetic tests can also be used to discover genetic defects at an early stage in order to provide appropriate care and facilitate selective breeding programs for domestic dogs. Mitochondrial genetics are inherited entirely from the maternal line; therefore, mtDNA analysis can be performed prior to breeding to ensure the health of descendants.

From another perspective, the application of this method indicates the possibility of analyzing the DNA of other types of dogs using canine fecal samples, and it may be possible to study the epidemiology of stray dogs through fecal DNA analysis. The increase in the number of stray dogs is a public health concern in many countries. This concern emanates from the fact that stray dogs and cats usually carry infectious diseases such as parvovirus, distemper, and rabies, which can be transmitted to humans through human contact [19,20]. In parallel with controlling stray dog populations, monitoring the health epidemiology of stray dogs in the community by collecting fecal samples from such dogs and collecting fecal DNA could be a potential measure. Analysis of the diverse DNA present in fecal samples could aid in the identification of the genetic material of viral, bacterial, and parasitic pathogens and identify infections that these animals may have. In this way, zoonotic diseases in stray dogs in public spaces can be monitored, and prevention and control measures can be rapidly implemented. Such measures would represent a means of not only protecting human health, but also improving the welfare of this venerable friend of man, the animal. Fecal DNA analysis across different geographic regions is also useful for epidemiological surveillance, revealing patterns, trends, and emerging threats, and allowing for a timely response. Fecal DNA analysis thus has the potential to be part of a comprehensive strategy to protect the health and welfare of both humans and domestic and stray dogs. Therefore, in addition to mtDNA analysis, as employed in the present study, the authors of future studies will be able to reveal in depth the usefulness of fecal samples by employing NGS to quantify the presence and proportion of DNA in samples obtained from various species.

It is recognized that the mitochondrial genome in mammalian cells is inherited from the maternal line [21]. The complete mitochondrial DNA sequences of a mother and her offspring exhibit a perfect match across their entire length, with the exception of instances where genetic disease is present. This finding was also corroborated in our previous research [10]. Consequently, the method used in the present study is not only capable of identifying individual dogs, but also tracing the blood relatives of dogs over many generations. Moreover, even in the absence of DNA sequence reference data, it is feasible to ascertain the presence of blood relatives if the DNA of the mother or siblings is available. In light of these considerations, it may also be feasible to address challenges at breeding facilities. For instance, at dog breeding facilities, there are instances where the maternal lineage of a puppy is uncertain, such as with mistaken puppies. In such cases, this method can be employed to ascertain the maternal lineage non-invasively. With such potential applications in mind, the method may prove beneficial in enhancing animal welfare in the future.

There is an interesting report on canine feces testing: in 2024, in Bolzano, northern Italy, the city’s police ordered all local dog owners to have their dogs’ fecal DNA tested in order to tackle the issue of dog feces left on the streets [22]. This process facilitated the identification and fining of owners who had not picked up their dogs’ feces. The DNA test used in the above study appears to involve the use of a method to detect short tandem repeats (STRs); however, our method of decoding the entire mtDNA may also be applicable. Each test appears to have its advantages and disadvantages. Our method involves the use of NGS, which facilitates the sequencing of the mtDNA of hundreds of dogs at a time and enables high throughput. Furthermore, even if the DNA sequence of the target dog is not registered, it is possible to trace the blood relationship, which may lead to the identification of the owner. However, if the number of samples is small (e.g., less than 10), the cost per sample increases significantly. In addition, advanced skills in NGS and bioinformatics analysis are required. STR-targeted tests are simple, and the results can be analyzed at low cost even with a small number of samples; however, they cannot identify dogs if their DNA sequences have not been registered. Thus, although each test has its advantages and disadvantages, using the two tests in different situations would help to obtain accurate evidence and achieve identification of the owner.

Another unique feature of the present study is the successful preparation of automated amplicon DNA libraries. DNA library preparation, when performed by human handlers, requires a high degree of skill and long hours of work. Therefore, there are significant barriers for beginners to perform this experiment. To solve this problem and compile the DNA library with simple operations, we used an experimental robot, LabDroid “Maholo” [23]. Our study likely represents the first attempt to use LabDroid “Maholo” in veterinary medicine and animal welfare. The above robot was created by a Japanese venture company [23] and specializes in molecular biology, biochemistry, and cell biology experiments. In recent years, the robot has been able to automatically create pluripotent stem cell- (iPSC) [24], and iPSC-derived retinal pigment epithelial (iPSC-RPE) cells [25,26], and the LabDroid innovation is currently being conducted in Japan. In addition, the robot’s user-friendly graphical interface allows researchers to create their own experimental programs from scratch. We successfully used this functionality to create an original amplicon DNA library. Experiments using the robot have also resulted in a significant reduction in operator time and thus a significant reduction in researcher labor. We hope to utilize this robot in the future for veterinary tests that require complex processes, thereby contributing to improvements in veterinary medicine and animal welfare.

In recent times, SNP (single nucleotide polymorphism) chips have been made available by major companies for the purpose of deciphering the genotype profiles of dogs. This method is capable of deciphering specific bases within the whole genome, thereby enabling the identification of individuals and their respective breeds. It is also likely to prove useful in the diagnosis of the mitochondrial diseases. Conversely, as this method is designed for the specific deciphering of bases, it is unable to accommodate unknown mutations. Pathological mutations or defects in mammalian mtDNA that involve amino acid substitutions are thought to occur randomly due to ROS (reactive oxygen species) [27,28]. As the mutations are not limited to specific bases, it is necessary to decode the whole mtDNA for detailed differential diagnosis. In consideration of the pathomechanism, it would be recognized that our method is superior to the SNP chips. The comprehensive nature of our method allows for the examination of the whole length of mtDNA, facilitating the acquisition of detailed sequence information encompassing not only the regions encoding enzymes associated with oxidative phosphorylation (OXPHOS) but also the non-coding regions. This comprehensive approach may prove advantageous in addressing the potential limitations of the SNIP chip. However, it may be somewhat inferior in terms of cost and ease of experimentation. Nevertheless, it is desirable to implement an examination method that matches the symptoms of the case, and this method would be a valuable contribution to the differential diagnosis of canine mitochondrial disease.

It should be noted that this study is not without limitations. This study employed fresh fecal samples that could be processed within one hour of defecation. Also, given the limited size of our dog community, we were finally able to use fecal samples from only two dogs. Ideally, it would have been preferable to analyze more than 10 dogs. However, when the results of the oral mucosal DNA and fecal DNA were compared, it was observed that the entire mtDNA sequence was identical. Additionally, in the experiment where LR-PCR was not performed (equivalent to whole fecal genome sequencing), no accurate sequence was obtained, and it was established as a negative control. In conclusion, despite the limitations imposed by the small sample size, the results were promising, and the negative control was also successful. Therefore, it can be reasonably asserted that the scientific validity of the results is assured. It remains unclear, however, whether the same method can be applied to degraded samples. For example, it is unclear whether the same results as in this study can be obtained when using feces that have been left at room temperature, in the hot sun, or in high humidity for a long period of time. This is a topic that we intend to pursue further in future research.

## 5. Conclusions

In conclusion, the results of our study show that the whole mtDNA sequence can be determined using fecal DNA from samples from domestic dogs with automated library preparation on the LabDroid “Maholo” and using NGS. Our findings suggest that feces can be adapted for mitochondrial disease, and their use in testing could aid the future medical care and welfare of domestic dogs.

## Data Availability

Data are contained within the article and Supplementary Materials. In addition, the raw data from NGS (FASTQ, BAM, and BCL files) can only be distributed to individual researchers if they are used for research purposes.

## Supplementary Materials

The following supporting information can be downloaded at: https://www.mdpi.com/article/10.3390/ani14192872/s1. Table S1: Sequences of the primer pairs and target location; Data S1 and S2: FASTA files of the whole mtDNA sequence determined using fecal DNA obtained from the sample of the two dogs.
